# An observational study on rhabdomyolysis in the intensive care unit. Exploring its risk factors and main complication: acute kidney injury

**DOI:** 10.1186/2110-5820-3-8

**Published:** 2013-03-14

**Authors:** Esmael El-Abdellati, Michiel Eyselbergs, Halil Sirimsi, Viviane Van Hoof, Kristien Wouters, Walter Verbrugghe, Philippe G Jorens

**Affiliations:** 1 Department of Critical Care Medicine, Antwerp University Hospital, University of Antwerp, Wilrijkstraat 10, Edegem, B-2650, Belgium; 2 Department of Clinical Chemistry, Antwerp University Hospital, University of Antwerp, Edegem, Belgium; 3 Department of Biostatistics, Antwerp University Hospital, Edegem, Belgium

**Keywords:** Rhabdomyolysis, Intensive care unit–ICU, Creatine kinase, Creatine phosphokinase, Myoglobin, Serum myoglobin, Urinary myoglobin, Acute kidney injury

## Abstract

**Background:**

Because neither the incidence and risk factors for rhabdomyolysis in the ICU nor the dynamics of its main complication, i.e., rhabdomyolysis-induced acute kidney injury (AKI) are well known, we retrospectively studied a large population of adult ICU patients (n = 1,769).

**Methods:**

CK and sMb (serum myoglobin) and uMb (urinary myoglobin) were studied as markers of rhabdomyolysis and AKI (RIFLE criteria). Hemodialysis and mortality were used as outcome variables.

**Results:**

Prolonged surgery, trauma, and vascular occlusions are associated with increasing CK values. CK correlates with sMb (*p* < 0.001) and peaks significantly later than sMb or uMb.

The logistic regression showed a positive correlation between CK and the development of AKI, with an OR of 2.21. Univariate logistic regression suggests that elevations of sMb and uMb are associated with the development of AKI, with odds ratios of 7.87 and 1.61 respectively. The ROC curve showed that for all three markers a significant correlation with AKI, for sMb with the greatest area under the curve. The best cutoff values for prediction of AKI were CK > 773 U/l; sMb > 368 μg/l and uMb > 38 μg/l respectively.

**Conclusions:**

Because it also has extrarenal elimination kinetics, our data suggest that measuring myoglobin in patients at risk for rhabdomyolysis in the ICU may be useful.

## Background

Rhabdomyolysis is a syndrome that is characterized by the disintegration of striated muscle and the leakage of intracellular muscular components into the blood and urine. It results in electrolyte disturbances and an elevation in the levels of sarcolemmal proteins, such as creatine kinase (CK) and myoglobin in body fluids [1,2].

The presentation of this multifactorial and multicausal syndrome varies from an asymptomatic but detectable elevation of CK and myoglobin in blood to a life-threatening condition with fulminant acute renal failure. The ability to predict rhabdomyolysis-induced acute kidney injury (AKI) is critical, because rhabdomyolysis is thought to be one of the leading causes of AKI. Indeed, 10–50% of patients with some degree of marked rhabdomyolysis develop AKI, and it has been suggested that rhabdomyolysis contributes to 5–25% of all cases of AKI [3].

The pathophysiology of rhabdomyolysis-induced AKI is believed to be triggered predominantly by myoglobin, which may cause renal dysfunction by direct renal cytotoxicity after reaching the tubules [4], vasoconstriction and tubular obstruction [1,5]. CK is the marker most commonly used to guide the diagnosis and therapy in daily clinical practice. Its half-life is 1.5 days [3]. In contrast, myoglobin is cleared much more quickly (half-life 2–3 hours), and clearance also occurs outside of the kidney [1]. The CK level that is associated with an increased risk of AKI has not been pinpointed, but values up to 5,000 U/L have been reported to increase the risk of rhabdomyolysis-induced AKI [6], especially when acidosis is present concomitantly [2,7]. Because most rhabdomyolysis studies are retrospective and involve small sample sizes, the exact role of myoglobin in the progression of the condition and the levels that lead to renal toxicity in rhabdomyolysis are still unclear [1,8-10].

Critically ill patients admitted to the intensive care unit (ICU) are at particular risk for rhabdomyolysis. Indeed, many of the known risk factors for rhabdomyolysis, including hypotension [1], trauma [6], electrolyte disturbances [11], drug abuse [12] and sepsis [13], occur commonly and often concomitantly in patients admitted to the ICU. Therefore, we studied a large number of patients in our ICU in whom serum CK and myoglobin (sMb) values as well as urinary myoglobin (uMb) levels were available. This large database allowed us to evaluate rhabdomyolysis risk factors in addition to the pathophysiological roles and predictive values of CK and myoglobin in the most important rhabdomyolysis-induced complication in the ICU environment: AKI.

## Methods

### Patients

This study was a single-center, retrospective, observational cohort study in the ICU of Antwerp University Hospital (Edegem, Belgium), the tertiary referral centre of the University of Antwerp. All adults who were admitted to our intensive care unit (which cares for both medical and surgical patients) during a period of 15 months (June 2008 to September 2009) were eligible for enrolment and retrospective evaluation. Because we sought cases of real rhabdomyolysis, we excluded patients admitted with elevated CK levels due to a myocardial infarction or acute coronary syndrome (ACS). Patients with a cerebral vascular infarction (CVA) or bleeding also were excluded, because damage to cerebral tissue can give rise to plasma CK-BB isoenzyme levels. Moreover, patients on chronic dialysis were excluded, because the outcome measures of AKI could not be objectively assessed.

### Procedures and data collection

This observational study without any specific intervention was reviewed and approved by the hospital’s Institutional Review Board (Reference number EC110219), and all data were anonymously processed. Informed consent was not required, because this was not an interventional study; however, all patients and families were informed on the admission leaflet that anonymous data may be used for academic research. In addition to serial biochemical and hematological laboratory data, several pieces of data were obtained for each patient. In our center, baseline muscular injury markers (CK, sMb, and uMb) are measured on admission to assess the breakdown of striated muscle, and troponin-I to assess cardiac muscle damage. Myoglobin levels in serum and urine (reported as μg/l) are determined with on immunoassay on an Access 2 immunoassay from Beckman Coulter (Analis, Suarlée, Belgium) using reagents of the same manufacturer. The baseline serum creatinine value was the creatinine obtained on admission in the ICU; the peak creatinine value was considered as the highest level during the ICU stay and therefore compared with the serum creatinine on admission.

Creatinine, CK, and Mb were always measured at the same time point, at least daily. If oliguria was present, uMb could of course not be measured. The uMb level in these patients was considered as missing value and not included in the statistical analysis.

In our ICU, blood is obtained in our patients on admission and at least daily (6 am) for the subsequent 72 hours unless the patient is discharged or deceased prior to that time. If CK is at least “moderately” elevated after 72 hours (CK level > 1,000 U/L), markers of muscular injury are usually followed daily until normalization of CK below the 1,000 U/l level. The following data were obtained: admission and maximum laboratory values that are routinely assessed daily in the ICU; demographic and clinical information, including age, gender, vital parameters (mean blood pressure and Pa0_2_ on admission); length of stay at the ICU and information necessary to determine the severity of organ failure, including the SOFA score (Sequential Organ Failure Assessment score [14]). On this scale, higher scores indicate more severe illness and a higher number of therapeutic interventions. Moreover, the inotropic index as a validated marker for the degree of hemodynamic support was calculated for each individual patient [15].

We systematically looked for risk factors for rhabdomyolysis as reported in the literature. Rhabdomyolysis is most commonly caused by direct physical trauma to skeletal muscle as a result of crush injury, extreme exercise, or seizures. Muscular ischemia is associated with rhabdomyolysis, most likely due to reperfusion injury after the restoration of adequate blood flow. Other causes include hereditary disorders, such as carnitine palmitoyltransferase II deficiency, malignant hyperthermia, and inflammatory disorders, such as dermatomyositis and polymyositis [1]. Prolonged surgery was arbitrarily defined as surgery for longer than 6 hours of duration, before ICU admission. Risk factors were considered relevant if they had occurred in the 24 hours before admission to the ICU.

Admission for fulminant rhabdomyolysis has been associated with the use of medication and illegal drugs [1]. HMG-CoA reductase inhibitors, fibrates, and diuretics are the agents most commonly associated with drug-induced rhabdomyolysis, and we evaluated the patients for systemic evidence of these medications [1,16,17]. We also screened for the use of toxic substances including ethanol, cocaine, and amphetamine, because these also are important causes of rhabdomyolysis [1,12,18,19]. The abuse of ethanol is specifically asked to every patient or his or her family upon admission in our ICU.

Numerical data were extracted from the patient data management system (PDMS system, IMD soft, Metavision) by a senior ICU staff member who was blinded to the patient’s rhabdomyolysis status. All other data were retrieved using an electronically prepared data acquisition form. These data were reviewed for missing information, logical errors, insufficient detail, or the need for additional queries and were analyzed by statistical software (Statistical Package for the Social Sciences, SPSS Inc., version 19). Patients were excluded from the analysis if the myoglobin values were not available.

### Specific outcome measures of renal failure

Complications and outcomes (AKI, need for dialysis and mortality) were recorded for each patient. AKI was defined using the RIFLE criteria with a minimum of 50% increase over baseline creatinine values. The acronym RIFLE stands for the increasing severity classes, risk (R) = 1, injury (I) = 2, and failure (F) = 3, and the two outcome classes, loss (L), and end-stage kidney disease (E). These criteria have been validated for ICU patients, such as those in our sample [20].

### Statistical analysis

All analyses were done in SPSS 19 (IBM, New York) and SAS 9.2 (SAS Institute, Cary, NC). Data are presented as median values with their lower and upper quartiles. Because CK, sMb, and uMb turned out to be log-normally distributed, log-transformations of these parameters will be used in all analyses.

The CK values were divided in four subgroups: 1) normal values with a maximum of 170 U/L; 2) between 170–1,000 U/l (moderately elevated up to more than five times the cutoff value); 3) between 1,000–5,000 IU/l; and 4) >5,000 U/L. These values were chosen because many clinicians use five times the upper limit of normal CK (approximately 850–1,000 U/l) as a reference point [21], whereas CK values of 5,000 U/L and higher are thought to be associated with an increased risk of rhabdomyolysis-induced AKI [6]. The subclass of values >5,000 was not divided further as hardly any patients presented with values >10,000 IU/l.

Patient characteristics are compared between these four CK groups by means of Kruskal-Wallis tests with subsequent post hoc analysis and Dunnett’s correction for multiple testing. For categorical variables, chi-square testing was used.

CK, sMb, and uMb are compared between RIFLE groups using ANOVA and post hoc analyses on the log-transformed values. Correlations between CK peak, sMb peak, and uMb peak, and their timing are assessed with Spearman correlation coefficients. Wilcoxon signed-rank tests were used to detect differences in timing.

Separate multiple logistic regression models were build to predict the development of AKI, with CK, sMb, and uMb as explanatory variables, taking into account other risk factors: baseline mean blood pressure, creatinine, CK, sMb, uMb, arterial Pa0_2_ on admission, lactate, osmolarity, SOFA, use of loop diuretics, presence of sepsis, hours of surgery exceeding 6, muscle ischemia, trauma, inotropic index, and the need for mechanical ventilation. For each of these models, ROC curves and their area under the curve are studied to evaluate the diagnostic performance of CK, sMb, and uMb. Cutoff values for CK, sMb, and UMb were calculated based on the ROC curve corresponding to the simple logistic regression models. With these cutoff values, a new multiple logistic regression model is built to study the effect of combined elevation (above cutoff) of CK and sMb.

Statistical significance was considered at the level of *p* < 0.05 for all comparisons.

## Results

### Global patient characteristics

A total of 2,699 adult patients were admitted to the ICU during the study period, and 1,769 patients were eligible for further evaluation. After the omission of patients with ACS (n = 179), CVA (n = 113), or on chronic dialysis (n = 40), the most common reasons for exclusion were missing myoglobin levels in either serum or urine. None of the included patients had received forced diuresis, mannitol, or bicarbonate as a preventive measure when CK elevation was seen. Table 1 displays the patient characteristics for every subgroup of CK values.

**Table 1:** Patient characteristics and outcomes in the ICU

**Creatine kinase (U/L)**	**<170**	**170–999**	**1,000–5,000**	**>5,000**
Count (n)	398	1029	283	59
Gender: men (%)	212 (53.9%)	676 (66.3%)	192 (68.6%)*	45 (77.6%)*
Age	65 (53–73)	66 (55–74)	62 (50–71)	63 (46–71)
ICU stay (days)	2 (1–6)	2 (1–4)	3 (1–9)*	6 (3–14)*
SOFA	5 (2–8)	6 (4–9)	7 (4–10)*	9 (5–13)*
AKI (%)	56 (14.1%)	181 (17.5%)	83 (29.3%)*	25 (42.4%)*
Hemodialysis (%)	34 (8.5%)	56 (5.4%)	33 (11.7%)	25 (42.4%)*
Mortality (%)	35 (8.8%)	51 (5%)	33 (11.7%)	16 (27.1%)*

Data are presented as median (Q1 - Q3).

AKI, acute kidney injury; ICU, intensive care unit.

*p < 0.05 compared with the group with normal CK levels (<170 U/L).

### Outcome

A total of 342 patients (20.4%) had CK values greater than 1,000 U/L. The SOFA score and outcomes (incidence of AKI, hemodialysis, and mortality) were significantly associated with increasing CK category (Table 1). In patients with highly elevated CK values (defined as >5,000 IU/l), hemodialysis was necessary in up to 42.4% of these patients and a mortality rate of 27.1% was observed in this group.

The etiologies of the observed CK elevations are displayed in Table 2. There were significant associations between CK levels greater than 5,000 U/L and recent (orthopedic) surgery. Trauma, resuscitation, compartment syndrome, and prolonged surgery for more than 6 hours also were observed more frequently in the same subgroup group with CK levels >5,000 U/L (Table 2).

**Table 2:** Etiology of elevated CK values in the ICU

**CK (U/L)**	**<170 (%) (n = 398)**	**170–999 (%) (n = 1029)**	**1,000–5,000 (%) (n = 283)**	**>5,000 (%) (n = 59)**
**Recent surgery**	39.5	84.1*	78.1*	59.4*
Cardiac	5.5	54.9*	31.4*	8.4
Abdominal	12.4	21.8*	27.8*	22.2
Vascular	6.6	5.8	5.1	16.7
Orthopedic	1	2.3	5.2*	12.9*
Thoracic	2.9	20*	19.1*	1
Neurologic	12.2	13.4	10.4	3.7
Other	3.1	2.8	3.1	1.8
**Muscle ischemia**	9.5	12.9	19.1*	49.9*
Arterial occlusion	5	3.7	2.6	9.3
Venous occlusion	1.6	2.4	0.5	0
Compartment syndrome	0	1.1*	1*	9.3*
Immobilization	1.6	1.5	2.6	3.7
Prolonged surgery	1.3	4.5*	12.9*	25.9*
Resuscitation	5.3	6.3	9.3*	11.1*
**Trauma**	2.4	7.5*	13.9*	16.7*
Blunt trauma	2.1	7.1*	13.4*	14.8*
Penetrating trauma	0.5	1.1	1	1.9
**Infectious**	48.8	52.4	51	42.6
Severe sepsis and septic shock	10.6	9.7	16*	20.4
Necrotizing fasciitis	0.3	0.4	1	1.9
Gram-positive infection	12.5	8.6	13.4	16.7
Gram-negative infection	25.7	18.8	17	25.9
**Use of drugs and toxins**	4.1	5.8	4	5.6
Ethanol	0.8	1.3	1.5	1.9
Cocaine	0.3	0.4	0.5	0
Heroine	0	0.4	0.5	0
Other/unknown	3	3.7	1.5	3.7

Data are presented as %.

*p < 0.05 (statistically significant compared with the CK level <170 U/l).

Immobilization = before admission; Prolonged surgery = surgery for more than 6 hours of duration.

All risk factors were considered relevant if they had occurred in the 24 hours before admission to the ICU.

Table 3 displays the mean peak CK, sMb, and uMb values with their corresponding *p* values for the different RIFLE groups. This table shows that patients who later develop AKI (RIFLE criteria 2 and 3) have significantly higher uMb, sMb, and CK values compared with RIFLE criterion 0. Our results also show a positive correlation between sMb and CK (r = 0.714, *p* < 0.001), but CK peaked significantly later than sMb (72 hours ± 145 SD vs. 30 hours ± 87 SD, *p* < 0.001) and uMb (72 hours ± 145 SD vs. 33 hours ± 77 SD, *p* < 0.001) values. There was a positive correlation between the timing of the CK peak and sMb peak (r = 0.304, *p* < 0.001) and between the timing of the CK peak and the uMb peak (r = 0.239, *p* < 0.001).

**Table 3:** Peak levels of CK and Mb according to the occurrence of acute kidney injury (RIFLE criteria used)

**RIFLE-criteria**	**None**	**Risk**	**Injury**	**Failure**
**Number (%)**	1,440 (81.4%)	171 (9.7%)	75 (4.2%)	83 (4.7%)
**CK (U/l)**	433 (186–744)	474 (257–869)	669 (439–1415)*	873 (410–2669)*
**sMb (μg/l)**	228 (107–444)	362 (158–764)*	617 (204–1382)*	740 (255–2349)*
**uMb (μg/l)**	9 (9–66)	17 (9–372)*	11 (9–341)*	58 (9–431)*

Data presented as median (Q1 - Q3).

*p < 0.05 compared with the group with RIFLE = 0, test on log-transformed CK, sMb, and uMb.

sMb, serum myoglobin; uMb, urinary myoglobin; CK, creatine kinase.

RIFLE : R(isk) = 1, I(njury) = 2, F(ailure) = 3, L(oss), E(nd stage).

The logistic regression model with log-transformed CK values in Table 4 showed a positive correlation with the development of AKI, with an odds ratio (OR) of 2.21 (confidence interval (CI) 1.45–3.38, *p* = 0.0002). Elevations of sMb and uMb are associated with the development of AKI, with ORs of 7.87 (CI 4.6–13.85, *p* < 0.0001) and 1.61 (CI 1.21–2.13, *p* = 0.001) respectively.

**Table 4:** Odds ratios for the development of acute kidney injury

	Models corrected for known risk factors*				
		**95% conf int**			**Area under**
	**OR**	**Lower**	**Upper**	**P value**	**ROC Curve**
**Log10 Creatine kinase**	**2.21**	**1.45**	**3.38**	**0.0002**	**0.759**
**Log10 Serum Myoglobin**	**7.87**	**4.60**	**13.85**	**< 0.0001**	**0.790**
**Log10 Urinary Myoglobin**	**1.61**	**1.21**	**2.13**	**0.0010**	**0.766**
CK 170 - 1000 U/L**	**1.42**	**0.84**	**2.45**	**0.1979**	
CK 1000 - 5000 U/L**	**2.56**	**1.27**	**5.21**	**0.0087**	
CK > 5000 U/L**	**5.12**	**1.84**	**14.29**	**0.0018**	

*Risk factors at baseline: mean blood pressure, creatinine, CK, sMb, uMb, PaO_2_, lactate, osmolarity, SOFA, use of loop diuretics, sepsis, hours of surgery > 6, inotropic index, mechanical ventilation, trauma and muscle ischemia.

**The different CK categories are compared with reference CK < 170 U/L.

When omitting the patients who died within 24 hours (n = 29) or 48 hours (n = 39) after admission in the ICU, neither the OR nor the area under the ROC curve changed. Moreover, this also was true when correcting the model for those patients discharged from the ICU within 24 hours (n = 676) and 48 hours (n = 938) respectively (data not shown). Only when performing the subgroup analysis by omitting patients hospitalized up to 24 hours (not 48 hours) in the ICU, the correlation between the development of AKI and uMb disappeared (data not shown).

**Figure 1:**
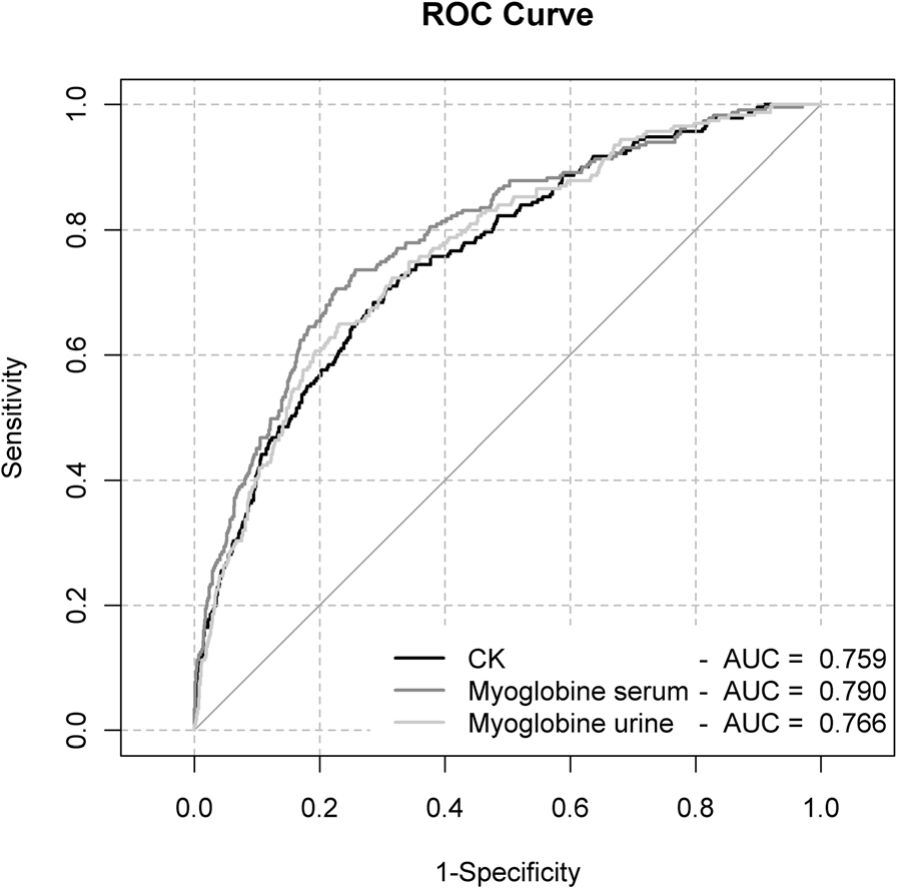
**ROC curve: predictive value of creatine kinase, serum and urinary myoglobin.** All three markers show significant correlation with acute kidney injury. Serum myoglobin has clearly the best predictive value. CK, creatine kinase; sMb, serum myoglobin; uMb, urinary myoglobin; cat, categorical (CK <170 U/L, CK 170–1,000 U/L, CK 1,000–5,000 U/L and CK >5,000 U/L).

Although for all three parameters (CK, sMb, uMb) there is a significant correlation with AKI, ROC curves (Figure 1) show that sMb has the greatest area under the curve and can therefore give the best prediction for AKI.

The best cutoff values for prediction of AKI, based on individual ROC curves, were CK > 773 U/l, sMb > 368 μg/l, and uMb > 38 μg/l, respectively. Table 5 displays the results of combining the cutoff values for CK and sMb in an additional logistic regression model, taking into account the other risk factors. It clearly shows that an elevated CK value alone, with sMb below the cutoff, does not lead to an increased risk for AKI (*p* = 0.8818). On the other hand, an elevation in sMb > 368 μg/l significantly increases the risk of AKI, regardless of CK elevation (OR respectively 4.3 and 5.1, *p* < 0.0001).

**Table 5:** Odds ratios for the development of acute kidney injury when CK and/or sMb are above their cutoff values (based on ROC curves)

	Model corrected for known risk factors*			
		**95% confidence interval**		
	**OR**	**Lower**	**Upper**	*p *value**
**CK > 773, sMb ≤ 368**	**1.06**	**0.45**	**2.27**	**0.8818**
**CK ≤ 773, sMb > 368**	**4.29**	**2.71**	**6.8**	**<0.0001**
**CK > 773, sMb > 368**	**5.11**	**3.03**	**8.63**	**<0.0001**

*Risk factors at baseline: mean blood pressure, creatinine, CK, sMb, uMb, PaO_2_, lactate, osmolarity, SOFA, use of loop diuretics, sepsis, hours of surgery > 6, inotropic index, mechanical ventilation, trauma and muscle ischemia.

**p value compared with the reference group CK ≤ 773 U/l and sMb ≤ 368 μg/l.

## Discussion

A limited number of observational and retrospective studies have examined the occurrence of rhabdomyolysis in critically ill patients in either the ICU [6,10] or the emergency department [22]. De Meijer and coworkers reported in another retrospective study that severe rhabdomyolysis, defined primarily on the basis of elevated serum CK levels of even greater than 10,000 U/l at some time during hospitalization in the ICU, was observed in 71 of 7,500 patients admitted over a long period [10]. Our observational study also clearly shows that the biochemical hallmarks of rhabdomyolysis (elevated levels of CK and/or myoglobin) are frequently observed in patients admitted to the ICU. The underlying causes of severe rhabdomyolysis have reported as being highly variable: ischemia by vascular obstruction and trauma (not commonly seen), sepsis and heatstroke/hypothermia in three patients each (11.5%) and hyponatremia in a single patient [10]. Vascular disease and trauma, known risk factors [3,7,10], also were determined to be contributing factors in our study. The causes of elevated CK and myoglobin levels in our study also were variable, but a primary cause seemed to be recent surgery (*p* < 0.001), especially prolonged surgery, in agreement with previous observations [23]. The other known reported etiologies of rhabdomyolysis, such as alcohol abuse [22], were not observed as important causal factors in our data. Of all known drugs reported to cause rhabdomyolysis, loop diuretics exposure before admission was the only drug associated with a marked CK elevation in our center. A limited number of patients were admitted to our ICU for alcohol or drug poisoning and polytrauma during this particular study period, which might have been responsible for the absence of these specific risk factors in our study.

Studies on rhabdomyolysis usually cover a wide disease spectrum with diverse etiologies, dissimilar diagnostic criteria for AKI and rhabdomyolysis, various methods, and differences in sample collection and decision cutoff values [24]. Furthermore, CK is generally considered the best overall parameter to determine the risk for development of its main complication AKI, whereas myoglobin is actually the main causative protein for developing AKI-associated rhabdomyolysis [1]. Moreover, most studies only analyze urinary myoglobin qualitatively using a dipstick method, which does not reliably differentiate between hemoglobin, myoglobin, and red blood cells [24]. Due to the retrospective nature of most studies on rhabdomyolysis in general and, especially, in the ICU setting, as well as the small sample sizes generally involved in these studies, the role of the muscular proteins in rhabdomyolysis and relevant serum values are still unclear. We therefore studied more than 1,700 patients to enable us to evaluate the role of CK, sMb, and uMb in rhabdomyolysis-induced AKI.

AKI has been defined differently in the studies that have investigated the relationship between CK and myoglobin levels and AKI. Some authors define AKI as a decline in renal function to such an extent as any form of renal replacement therapy is necessary [10]. Brown and coworkers defined AKI as a peak creatinine of 2.0 mg/dl [6], whereas we used the consistent and more recent RIFLE criteria with a minimum of 50% increase in baseline creatinine values [20]. Although previous studies [6,10] and reviews [2,3] suggest that a CK level of either >5,000 U/L or 10,000 U/L is associated with a higher rate of renal failure, we demonstrate by using a logistic regression model and ORs that a CK level of more than 1,000 U/L is associated with almost a threefold increased odds for developing AKI in the ICU compared with the group with normal CK values. Furthermore, our data indicate that a CK level of >5,000 U/L is even associated with an odds ratio of 5.1 for developing AKI.

Although myoglobin is thought as the single most important cause of rhabdomyolysis-induced AKI [2], only studies with small sample sizes are available to explore this hypothesis [24]. In our study, there is a strong correlation between sMb and CK. sMb peaks significantly earlier than CK (30 vs. 72 hours), which also is true for uMb (33 vs. 72 hours). These results are in agreement with previous observations that demonstrated a peak in myoglobin concentration before peaks in CK following muscular injury [8,9,25]. Myoglobin is not routinely assessed in all centers as it is at our site. The availability of serum myoglobin in our hospital as a more or less routine marker as well as the turnaround time of less than 90 minutes from taking the blood sample until the validated value is available is a rather unique situation compared with most other ICUs. Furthermore, myoglobin also is available as a point-of-care test with a turnaround time of less than 20 minutes in our emergency department.

The interpretation of results also is hampered by unpredictable renal and hepatic elimination of plasma myoglobin, and published correlation with the degree of myoglobinemia is poor. Therefore, although myoglobin is known to play a role in kidney injury, serum levels of CK are usually monitored to guide therapy [8]. We report that myoglobin has a strong correlation with AKI and CK in the ICU, and it peaks significantly earlier than CK. The quantitative evaluation of myoglobin is not overly expensive and can provide timely and valuable information regarding the development of AKI in the ICU setting. As other authors have suggested, the initiation of therapy could be guided by using serum myoglobin alone or in addition to CK [8,25,26]. In a small group of 30 ICU patients with massive rhabdomyolysis, receiver operating characteristic analysis showed that the AUC for blood myoglobin that predicted acute renal failure was 0.88, and the best cutoff value for blood myoglobin was 3865 μg/l [26]. The cutoff values in our study were 773 U/l for CK and 368 μg/l for sMb respectively. This much lower cutoff value for Mb can be explained by the much larger group in our study as well as the use of validated RIFLE criteria as opposed to creatinine change only. Because myoglobin is eliminated quickly and elimination occurs outside of the renal system [8,9,26], our data indicate that routinely measuring myoglobin on admission and, subsequently, in patients at risk of developing rhabdomyolysis in the ICU setting might be of clinical value.

There are some limitations to using myoglobin as a predictor of AKI. SMb and uMb are not routinely available and are only performed in many institutions when CK has been shown to be markedly elevated. In addition to the fast elimination kinetics, there is a large overlap of mean myoglobin values, which makes laboratory data difficult to interpret. However, this overlap also is present in the mean CK value in each different RIFLE group (Table 3). The mean values for CK and urinary myoglobin of the relatively more severe cases (RIFLE group 2 (I) and 3 (F) differ significantly from the non-AKI group. Nevertheless, the large standard deviation makes it difficult to interpret single case values in clinical practice. The risk profile of the patient and the ORs for CK, sMb, and uMb can help to evaluate these patients.

Although this is a large study on rhabdomyolysis in the ICU, some limitations should be taken into account. To ensure the quality of this retrospective observational study, some patients had to be excluded. Up to 38% of the patients in the primary study group were excluded because their admission diagnosis was known to cause CK elevations or based on the absence of complete myoglobin data, leading to a certain degree of bias. We are convinced that our data reflect “real life” in the ICU as excluding the patients who died soon after their admission to the ICU or who only stayed for a “short” period (up to 48 hours) had no influence on the analysis of the outcome parameters. Moreover, the kinetics of myoglobin release might vary between patients and myoglobin was only available at one time point each day: real peak values might therefore be missed as measuring the exact peak values might imply a more frequent sampling paradigm. Blood and urine sampling was performed daily at 24-hour intervals with some patients therefore having only two samples performed. This lends itself to the possibility of underestimating the peak serum myoglobin (i.e., marker may rise and fall between the sampling period) and furthermore the possibility of missing secondary peaks in the event of a secondary insult.

The contribution of certain risk factors is difficult to assess. Alcohol abuse is systematically questioned in our unit, but neither the determination of ethanol levels nor a drug screening is routinely performed upon admission, therefore certainly leading to an underestimation of their real contribution. In addition, we used the RIFLE criteria to define AKI. These criteria also are based on creatinine values. Creatinine itself is an intramuscular component that is associated with a more rapid increase in rhabdomyolysis-induced AKI than other causes of AKI [2]. However, the RIFLE criteria are validated in the ICU, which is relevant for our patient population [20]. Future studies therefore could use biomarkers of cellular kidney injury to look for early and subtle kidney damage.

This study was noninterventional and tried to predict which patients have an increased risk for developing AKI, but it does not suggest when and which therapy should be initiated. Therefore, there is a great need for prospective studies on this subject to evaluate the roles of CK, uMb, sMb, and the effect of therapy on preventing rhabdomyolysis-induced AKI. However if a prospective study was to be planned, myoglobin sampling ideally would be performed more frequently.

## Conclusions

Our study demonstrates that muscle injury or rhabdomyolysis is frequently observed in patients admitted to the ICU, and this seems to contribute to AKI. Previous surgery, and especially prolonged surgery, was recognized as the main causal factor. Moreover, sMb and uMb levels show significant positive ORs for predicting AKI and peak significant earlier than CK. These data, the underlying pathophysiological role of myoglobin, and the relatively low cost of evaluating myoglobin levels lead us to suggest that myoglobin could be assessed more routinely in the ICU in patients at risk for developing AKI. Further studies are necessary to evaluate the roles of CK, uMb, and sMb and the effect of therapy on preventing rhabdomyolysis-induced AKI.

## Abbreviations

AKI: Acute kidney injury; ICU: Intensive Care Unit; CK: Creatine Kinase; CK-BB: Brain isoform of creatine kinase; CK-MB: Cardiac isoform of creatine kinase; Mb: Myoglobin; sMb: Serum myoglobin; uMb: Urinary myoglobin; SOFA: Severity organ failure assessment; PDMS: Patient data management system; ACS: Acute coronary syndrome; CVA: Cerebral vascular accident; CI: 95% Confidence interval; OR: Odds ratio

## Competing interests

The authors declare that they have no competing interests.

## Authors’ contributions

Authorship credit is based on 1) substantial contributions to conception and design, acquisition of data, or analysis and interpretation of data; 2) drafting the article or revising it critically for important intellectual content; and 3) final approval of the version to be published. All authors meet conditions 1, 2, and 3. All persons designated as authors qualify for authorship, and all those who qualify are listed. Each author has participated sufficiently in the work to take public responsibility for appropriate portions of the content. EEA, ME, HS, and WV gathered the data. VVH performed the biomarker analyses. EEA, ME, HS, and KW executed the statistical analysis. EEA, ME, and HS drafted the manuscript. All authors read and approved the final manuscript.
